# Curious and Unusual Case of Phymosis

**Published:** 1859-12

**Authors:** W. H. Butler


ART. V. *Curious and Unusual Case of Phymosis. Complete Union of Prepuce and Glans.* By W. H. BUTLER, M. D.

THE patient, John C——, aged about forty, has been for three or more years troubled with difficulty in urinating, and for the last year his urine has come away in drops. About a year ago, he had complete retention of urine, which lasted twenty-four or thirty-six hours, accompanied with much pain. This was relieved by his own efforts, the urine ultimately coming away drop by drop; and this condition obtained till July of this year. On the 16th of that month the patient, having been unable to pass his water for forty hours, and experiencing acute pain, his cries being heard for a long distance, called in Dr. W. H. Newell, who asked my assistance. On examination, we found the bladder enormously distended, the fundus reaching to near the umbilicus. The penis seemed devoid of urethra—or the prepuce had agglutinated over it, and there was complete consolidation of prepuce and glans, not the least point appearing for the entry of the smallest-sized probe. After making an incision where the opening of the urethra ought to have been, we succeeded, after several minutes' trial, in introducing a very small, whalebone probe two inches within the urethra; and on its withdrawal, a minute stream of urine followed. This would cease in a few seconds, and require a repetition of the probing.

The stoppage being apparently due to the phymosis, an incision was made onto the glans, and a director forced between it and skin, and the prepuce slit up. Owing to the complete growth of the two, it required considerable force, and much difficulty was experienced in accomplishing it. As considerable time had been consumed, and the patient was enabled, by this operation, to pass his urine, it was deemed advisable to defer, till the next day, any further interference. On examination, the following morning, it was concluded to excise the foreskin, which was grown close to the glans. This having been done, complete relief was given to the constricted urethra, enabling the largest-sized bougie to enter with ease. One was left in the urethra to prevent union of the cut edges, and the patient put in bed. The next day he was about; and in two weeks after, when I heard from him, he was well. He passes his water in a full, large stream, and the excised part has healed kindly. We learned from the patient that he has begot children since this constriction has existed. We certainly will have a better chance for such accidents in the future.

